# An Evaluation of the Accuracy of Digital Models—An In Vitro Study

**DOI:** 10.3390/dj12100313

**Published:** 2024-09-29

**Authors:** Kinga Mária Jánosi, Diana Cerghizan, Eszter Elza Bai, Izabella Éva Mureșan, Alpár Kovács, Andrea Szász, Adrian Hulpe, Emese Rita Markovics, Krisztina Ildikó Mártha, Silvia Izabella Pop

**Affiliations:** 1 Faculty of Dental Medicine, George Emil Palade University of Medicine, Pharmacy, Science and Technology of Targu Mureș, 38 Gh. Marinescu Str., 540139 Targu Mureș, Romania; kinga.janosi@umfst.ro (K.M.J.); krisztina.martha@umfst.ro (K.I.M.);; 2 Private Practice, 540501 Targu Mureș, Romania; 3 Private Practice, 540044 Targu Mureș, Romania

**Keywords:** intraoral scanner, digital impression, digital dentistry, accuracy

## Abstract

Background: Intraoral scanning technology has opened new perspectives in dental practice, and combined with CAD/CAM technology, contributes significantly to fabricating high-quality prosthetic restorations. Our in vitro study aims to assess the accuracy of digital models obtained from one laboratory and two less commonly used intraoral scanners by conducting 3D measurements on the digital models obtained. Methods: An articulated simulator cast was used. Forty-eight scans were performed before and after tooth preparation with each scanner. The Zeiss Inspect software (Version: 2023.3.0.969) was used for measurements in sagittal and transversal planes. The obtained values were compared to reference values resulting from manual measurements. Results: Digital impressions provided discrepancies compared to the reference model. The lowest differences at the A2-L2 (the diagonal dimension of the models from the distal fossa of the second right maxillary molar and the maximum oral convexity of the artificial gingiva at the first left premolar) and the A1-B1 (transversal dimension of the model in the posterior area, from the right second molar’s occlusal central fossa to the left second molar central fossa) distances were obtained for the upper models, and at the a1-b1 distance for all the lower models, except the non-prepared models scanned with the intraoral scanners (the discrepancies were not statistically significant). The discrepancies increased with the distance from the starting point of the scan. Conclusion: The number and position of prepared teeth can influence the accuracy of the scans. Distortions can appear in the case of multiple preparations. The scanning protocol and calibration must be optimized for the highest accuracy. Furthermore, in vivo studies are necessary to evaluate the clinical applicability of these findings.

## 1. Introduction

Intraoral scanning technology has recently opened new perspectives in dental practice, helping to streamline and simplify prosthetic restorative treatments. Approaching patients digitally in combination with digital CAD/CAM technology [1] contributes significantly to the rapid, chair-side fabrication of high-quality prosthetic restorations.

Studies in the literature have demonstrated the clinical applicability of this method. Digital impressions are considered a good alternative to conventional impressions [2,3,4]. Most studies are based on research with high-cost, high-performance intraoral scanners, leaving a gap in information about less commonly used but cheaper scanners. Their advantages, like lower price, easy use, and simple software (without smile design possibilities or more complex procedures beyond digital impression-taking) make them accessible to clinicians when replacing conventional impressions. However, evaluating their accuracy for successful clinical application is essential.

The intraoral scanners facilitate diagnosis, allow measurements to be taken using digital software, and contribute to developing orthodontic, restorative, or implant-prosthetic treatment plans [5]. This technique has been used more frequently in recent clinical practice, with intraoral scanning becoming a popular alternative to more time-consuming impression techniques. This approach is effective for prosthetic restorations of up to ten elements and smaller edentulous spaces [6,7]. Several studies have indicated that intraoral scans offer a more accurate alternative to conventional impressions, particularly in cases where the edentulous space is relatively short and the scanning is extended to a single quadrant or a few abutments [8,9].

According to ISO 12836:201213, clause 3.6, accuracy describes the degree of agreement between a given measurement result and the actual value of the object to be measured. Accuracy is determined by two independent factors: Trueness and Precision [10]. Trueness is the average deviation of the measurement from the actual value [11]. Precision (reproducibility) is the standard value error of repeated measurements. It indicates the number of times the test procedure has been repeated under the specified conditions and the degree of agreement between the results obtained when the test is repeated several times under specified conditions [12,13]. These devices use complex optical technology to capture three-dimensional intraoral images. Using these images, the scanner software is able to create digital models for different dental use. Different non-contact optical technologies (triangulation, confocal microscopy, optical coherence tomography, interferometry, stereovision) are often combined to reduce noise during the intraoral scanning of surfaces with different reflectivity or transmittance (teeth, soft tissues, occlusal lines, various types of restorations) [14]. One of the most significant advantages of digital impressions is the elimination of the need to place the impression tray and different materials in the mouth during conventional impression-taking procedures, which is uncomfortable for patients. This advancement has significantly improved patient comfort during impression taking, thereby improving the overall dental experience associated with intraoral scanning [15,16]. In addition, the accuracy of these scanners in treatment planning and providing prosthodontic restorations has improved the quality of dental care and resulted in more accurate restorations and more satisfied patients during the digital workflow [17].

The development of intraoral scanners has been remarkable in recent decades. Manufacturers have responded to clinicians’ needs for more compact and user-friendly devices by developing higher-speed and more accurate scanners. The actual devices offer real-time, chair-side feedback, color scanning capabilities, and artificial intelligence-driven procedures, improving the scanning experience and ensuring optimal clinical outcomes. These developments represent the future of dentistry and promise a more efficient and effective way to deliver dental care.

The study aims to evaluate the accuracy of digital models obtained from one laboratory and two less commonly used intraoral scanners after performing three-dimensional measurements on the digital models obtained before and after tooth preparation, following the manufacturer’s recommended scanning path. Given the insufficient literature, this would provide clinicians with information about the accuracy of the scanners used to obtain digital impressions, an essential element for a successful prosthodontic workflow, especially in multiple prepared abutments. The null hypothesis was that there would be no statistically significant differences regarding the accuracy between the non-prepared digital models and those with multiple preparations obtained with laboratory and intraoral scanners; the manufacturer’s recommended scanning path would result in accurate digital models in the case of self-calibrating systems.

## 2. Materials and Methods

This in vitro study was approved by the Ethics Committee of the “George Emil Palade” University of Medicine Pharmacy, Science and Technology from Targu Mures (3074/12.04.2024).

### 2.1. Study Design, Sample Selection

The sample size for this study was determined using G*Power version 3.1.9.6. software (Franz Faul, Universität Kiel, Kiel, Germany). The calculations indicated that a minimum of 42 digital models per group (upper arch and lower arch) would be necessary; this size would provide greater than 95% power to detect significant differences, with an effect size of 0.80 at a significance level of α = 0.05.

This study used an articulated simulator cast with integral dental arches. Both the upper and lower dental arches were scanned forty-eight times. A total of 192 upper and 480 lower artificial teeth were prepared.

The procedures were conducted at the Department of Fixed Prosthodontics, Faculty of Dental Medicine of the “George Emil Palade” University of Medicine, Pharmacy, Science, and Technology from Targu Mures, between 10 February 2024 and 10 March 2024.

The in vitro study consisted of digital impressions of the simulator dental arches, obtaining S.T.L (Standard Tessellation Language) files with different scanners. New scans were performed after tooth preparations on the upper and lower dental arches with each scanner, followed by the dimensional comparison of the measurements performed on the obtained digital models. One experienced operator performed the scans and the tooth preparations to obtain comparable testing conditions and to reduce or avoid mistakes [18].

### 2.2. Scanning Procedures

Powder-free, color, intraoral scanners and a laboratory scanner (with powder) were used to scan the simulator cast before and after tooth preparation.

The Neway OpenTech laboratory scanner—NOT (Open Technologies Rezzato BS, Brescia, Italy)—was used for the most accurate scanning. This scanner, with a large scanning area and two color cameras, allows easy and intuitive color scanning with an accuracy of 5 µm (ISO 12836 and VDI/VDE 2634), a repeatability of 2 µm, and a speed of one arch scanning in about 14 s. The high scanning speed combined with the included and optional accessories (multi-bridge holder, Conceot Quad kit) contributes to an increased scanning performance. The open software (Scan Way, Version: 1.5) of the scanner processes the data obtained, allowing us to import or export data in STL, OBJ, OFF, or PLY formats. These are known and used formats in digital dentistry. This scanner, with constantly available automatic updates, requires periodical calibrations and utilizes a structured light source to scan.

The Virtuo Vivo intraoral scanner—VV (Dental Wings Inc., Montreal, QC, Canada)—and the Evo I.O. Scan Fussen—IOS (Fussen Technology Co., Shenzhen, China)—were used in this study for scanning the dental arches.

The Evo I.O. Scan Fussen S 600 is a calibration free scanner, with a built-in anti-fog function, a scanning accuracy of 25 µm (10 µm for single tooth restorations), a depth of field of 15 mm, and a scanning speed of 90 s per arch. Its scanning principle is triangulation, using the structured light source. The obtained data can be exported in STL, OBJ, and PLY formats.

The Virtuo Vivo scanner’s scanning technology is multi-module stereoscopy. Its scanning accuracy for the full-arch scanning is 21 µm with a 20 mm depth of field. The scanner allows for the color capturing of the scanned objects. The data can be exported as STL or PLY formats. 

For the upper arch, the scan direction was from right to left. The scanning started from the occlusal surface of the most posterior tooth of the right hemiarch on the simulator models, scanning consecutively from the occlusal surfaces of the artificial teeth. From the right canine, the scanning proceeded by moving the head of the scanner in an “S”-motion in a bucco-lingual direction. For the Virtuo Vivo scanner, this sequence was carried out starting from the canine, and for the Evo I.O. Scan Fussen S 600, from the lateral incisor.

In the lateral areas, the scanning of the occlusal surface continued towards to the last molar from the other side. The occlusal surfaces were scanned up to the last molar from the right, while maintaining a 45-degree inclination for the scanner head. Buccal surfaces were scanned consecutively by continuously moving the scanner head towards the last left molar. The scans were analyzed and missing or deficient areas were corrected.

In the case of the lower arch, the scanning direction was inverse, starting from the last left molar.

All the models were scanned before tooth preparation. After the preparation of the first series of artificial teeth (the premolars on both sides of the upper model, and all the teeth between the two second premolars on the lower models), new artificial teeth were placed on the initial model, which was rescanned. The new teeth were prepared, and a new scan was performed. This procedure was repeated 48 times on both models (upper and lower). The scans were obtained by the same experienced and trained operator who performed the tooth preparations.

### 2.3. Tooth Preparation

The teeth were prepared with a “heavy chamfer” finish line, following the general and specific tooth preparation principles.

The articulated simulator model was fixed in the Planmeca simulator (Planmeca Oy, Helsinki, Finland) from the Simulation Center of the Faculty of Dental Medicine from the University of Medicine, Pharmacy, Science and Technology “G. E. Palade” from Targu Mures. The preparation was carried out in the supine position of the patient, from the 12 o’clock position (ISO Standard 11226: “Ergonomics-Evaluation of static working postures”).

A medium-grained, round-end tapered diamond bur with a standard conicity of 3°, size 016, was used to create the equigingival finish line. The diameter of the active part of the bur was 1.1 mm. During the preparation, the bur was held parallel to the tooth axis, resulting in a minimal taper and a 0.5 mm width of the finish line. The premolars were prepared on the upper arch and all the teeth from the lower arch were prepared except for the molars.

### 2.4. Measurements/STL Files Comparison

To obtain reference values for model analysis, measurements (sagittal and transverse plane) were performed on the simulator model (reference values) by using the Burg Wachter Precise PS 7215 digital caliper (Burg-Wachter KG, Wetter (Ruhr), Germany) with a measuring accuracy of ±0.002 mm/m. A fine point Sharpie pen (Newell Brands Inc., Atlanta, GA, USA) with a tip diameter of 0.4 mm was used for marking the reference points used for the measurements.

**Figure 1:**
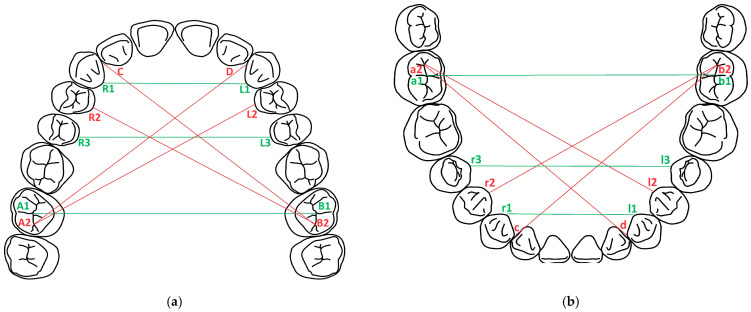
Reference points and distances in the two planes: (**a**) maxilla; (**b**) mandible. Red lines: diagonal distances, green lines: transversal distances.

The reference points were placed on both the upper arch and the lower arch (Figure 1):

- A1, B1, a1, b1: in the central fossa of the molars 17, 27, 47, 37;
- A2, B2, a2, b2: in the distal fossa of the molars 17, 27, 47, 37;
- C, D, c, d: at the tip of the artificial interdental papillae in the interdental spaces 12–13, 22–23, 42–43, 32–33;
- R1, R2, R3, L1, L2, L3, r1, r2, r3, l1, l2, l3: on the artificial gingiva at the maximum convexity of the oral cervical contour of the teeth 13, 14, 15, 23, 24, 25/43, 44, 45, 33, 34, 35;
- To obtain the values in the transversal plane, the following distances were measured: R1-L1, R3-L3, A1-B1, r1-l1, r3-l3, a1-b1;
- To obtain the values in the sagittal plane, the following distances were measured: A2-D, A2-L2, B2-C, B2-R2, a2-d, a2-l2, b2-c, b2-r2.

**Figure 2:**
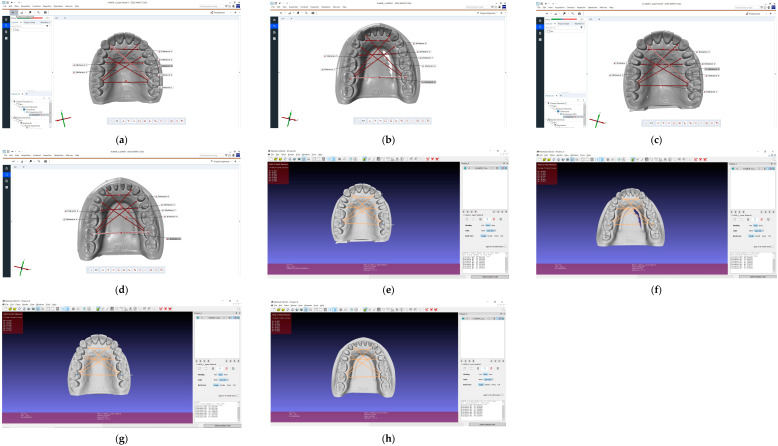
The measurements performed on digital models: (**a**) upper non-prepared model in Zeiss Inspect; (**b**) lower non-prepared model in Zeiss Inspect; (**c**) upper prepared model in Zeiss Inspect; (**d**) lower prepared model in Zeiss Inspect; (**e**) upper non-prepared model in MeshLab; (**f**) lower non-prepared model in MeshLab; (**g**) upper prepared model in MeshLab; (**h**) lower prepared model in MeshLab.

For the visualization of the landmarks in the measurement software, a very thin, fine-grained, needle diamond bur was used at the landmarks. The bur was held perpendicular to the tooth surface or the gingival surface. The simulator models were then scanned with these marks, which were retained during tooth preparation. The data obtained from the scanning of the models were exported in open STL format, which only allows a monochrome display of the models. Zeiss Inspect 2023 (Zeiss Quality Suite, Zeiss, North Maple Grove, MN, USA) and MeshLab 2023.12 (Visual Computing Lab, ISTI-CNR, Pisa, Italy) were used to analyze the models and to perform the measurements (Figure 2). The measurements were carried out in both planes (sagittal and transverse), as in the case of the reference model.

**Figure 3:**
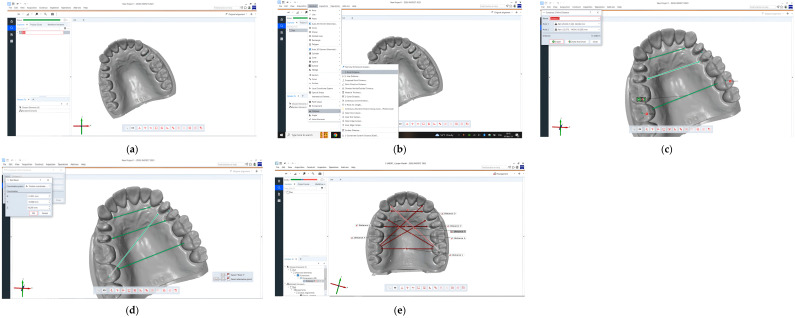
The measurements performed step-by-step in Zeiss Inspect software (Version: 2023.3.0.969): (**a**) importing the files in original alignment; (**b**) selecting the “Construct 2-point distance” from the toolbar; (**c**) lines drawn for transversal measurements; (**d**) line drawn for sagittal measurements; (**e**) reference lines with their length.

For the better precision of the digital measurements, the values from Zeiss Inspect software (Version: 2023.3.0.969) were calculated and evaluated only (Figure 3). These values were compared with the reference values of the physical models for each model and scanner.

### 2.5. Statistical Analysis

The statistical analysis in this study was performed using GraphPad Prism 10 for macOS version 10.2.3 (GraphPad Software, Boston, MA 02110, USA). The mean (M), median (Me), and standard deviation (SD) were calculated. The statistical significance was set at *p* < 0.05. Given the non-normal distribution of the data, as confirmed using the Shapiro–Wilk test, and the heterogeneity of variances indicated using Levene’s test, we employed the Kruskal–Wallis test for the initial analysis. This choice was guided by the test’s suitability for non-parametric data. After identifying significant differences, Dunn’s post hoc test was proposed to pinpoint specific group disparities. The Wilcoxon test was used to explore the differences between the measurements performed on virtual models and the reference model.

## 3. Results

The results of the descriptive statistics are shown in Table A1 and Table A2.

All measurements performed on the maxillary models, regardless of the scanner used or whether there were prepared teeth; these measurements exhibited extremely significant statistical differences (*p* < 0.0001) compared with the reference model.

The lowest discrepancies for VV were observed for A2-L2 in both cases (with and without preparations), the highest for B2-C for models without prepared teeth, and for A2-D for models with prepared teeth.

For NOT np, the lowest discrepancy was with A1-B1 and the highest with R1-L1; meanwhile, for NOT p, the lowest was with A2-L2 and the highest with A2-D.

**Figure 4:**
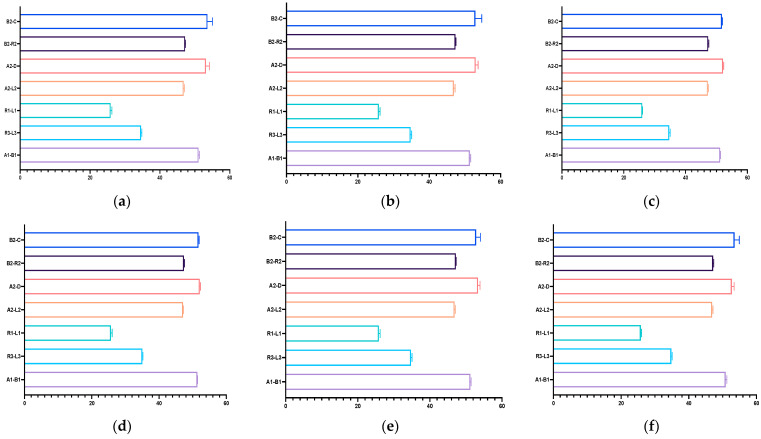
Graphical representation of the resulting measurements performed on the maxillary virtual models: (**a**) Virtuo Vivo scanner, non-prepared model; (**b**) Virtuo Vivo scanner, prepared model; (**c**) Neway Open Tech scanner, non-prepared model; (**d**) Neway Open Tech scanner, prepared model; (**e**) Evo I.O Scan Fussen scanner, non-prepared model; (**f**) Evo I.O Scan Fussen scanner, prepared model.

In the case of IOS with and without prepared teeth, the highest discrepancy was with B2-C, A2-D, and the lowest was with A1-B1, A2-L2 (Table 1, Figure 4).

**Figure 5:**
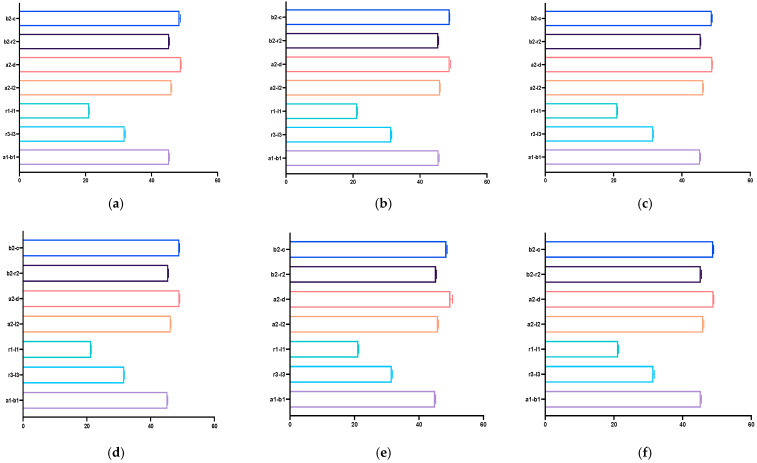
Graphical representation of the resulting measurements performed on the mandibular virtual models: (**a**) Virtuo Vivo scanner, non-prepared model; (**b**) Virtuo Vivo scanner, prepared model; (**c**) Neway Open Tech scanner, non-prepared model; (**d**) Neway Open Tech scanner, prepared model; (**e**) Evo I.O Scan Fussen scanner, non-prepared model; (**f**) Evo I.O Scan Fussen scanner, prepared model.

Most measurements performed on the mandibular models showed significant differences, except those carried out on the virtual models without prepared teeth, obtained after scanning with Virtuo Vivo and Evo I.O. Scan and comparing with the reference model. For values that did exhibit statistical differences, the lowest discrepancies were consistently observed for distances a1-b1. The highest discrepancy for VV np and NOT p was with r3-l3 and for the other virtual model with a2-l2 (Table 2, Figure 5).

**Figure 6:**
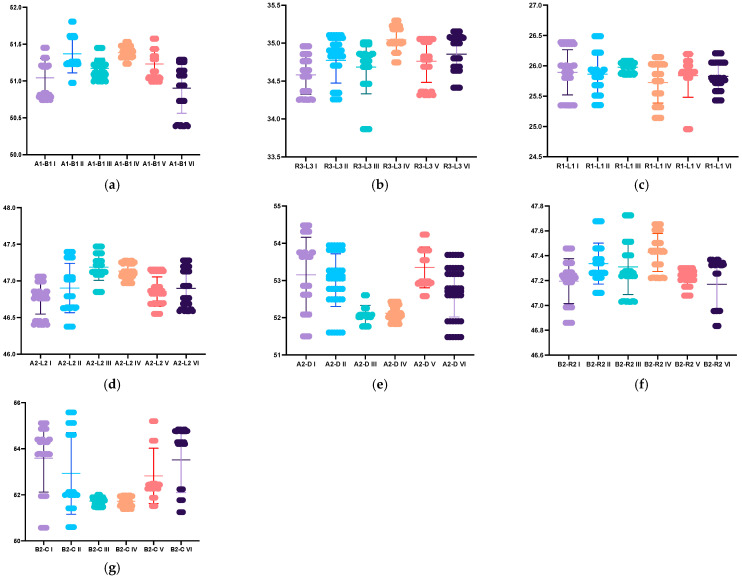
Graphical representation of the discrepancy between maxillary distances across the different used scanners and on the models with prepared or unprepared teeth: (**a**) A1-B1 distance; (**b**) R3-L3 distance; (**c**) R1-L1 distance; (**d**)A2-L2 distance; (**e**)A2-D distance; (**f**) B2-R2 distance; (**g**) B2-C distance. I: VV np, II: VV *p*; III: NOT np, IV: NOT np, V: IOS np, VI IOS np.

**Figure 7:**
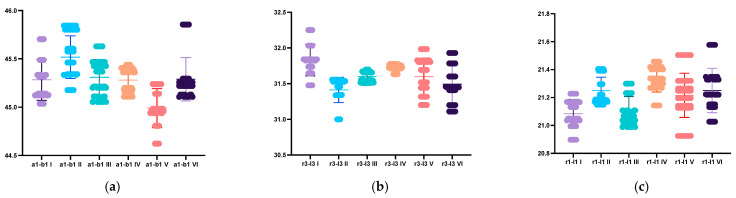

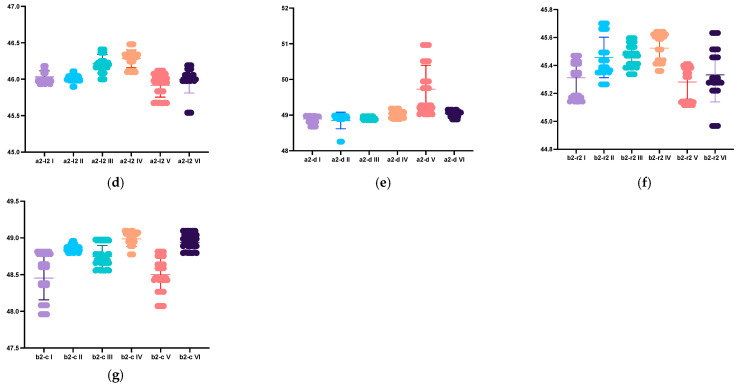
Graphical representation of the differences between mandibular distances across the different used scanners and on the models with prepared or unprepared teeth: (**a**) a1-b1 distance; (**b**) r3-l3 distance; (**c**) r1-l1 distance; (**d**) a2-l2 distance; (**e**) a2-d distance; (**f**) B2-R2 distance; (**g**) B2-C distance. I: VV np, II: VV p; III: NOT np, IV: NOT np, V: IOS np, VI IOS np.

The Kruskal–Wallis test revealed significant differences in the distribution of A1-B1 (*p* < 0.0001), R3-L3 (*p* < 0.0001), R1-L1 (*p* = 0.0041), A2-L2, A2-D, B2-R2, and B2-C (*p* < 0.0001) across the different used scanners and on the models with prepared teeth or unprepared teeth, suggesting variability in these parameters’ behaviors. The same pattern was observed across the mandibular measurements (Table A3 and Table A4; Figure 6 and Figure 7).

## 4. Discussion

The accuracy of the different scanners used to take digital impressions during prosthetic rehabilitation is a controversial topic in the literature. Therefore, different methods have been used to evaluate the accuracy of the digital models obtained using the scanners. Some of these methods are based on the accuracy of restorations fabricated on digital models obtained using direct or indirect scanning [19,20]. In this case, the errors that occur during scanning are difficult to distinguish from those that occur during the fabrication of restorations or from those that occur during the indirect digitization of models made from conventional impressions. The most-used methods are based on direct three-dimensional inspection using reference elements and a best fit alignment for comparisons on the surface of the reference elements during accuracy evaluation [21]. Research based on the assessment of accuracy using well-defined measurements requires specific knowledge, but also the ability to analyze statistical data obtained from comparing values from in vitro or in vivo measurements [22,23,24,25]. Determining trueness in vivo is difficult because the reference geometry is in the oral cavity and cannot be standardized and digitized directly to obtain an accurate reference. In vitro tests are easier to perform and provide a better insight into the accuracy of IOS, which is why they are good precursors to in vivo research [26,27]. Several recent studies have shown that the differences in accuracy and precision between in vivo and in vitro scans are minor [28,29].

This study investigated the discrepancy between the digital models obtained via two less commonly used intraoral scanners and a laboratory extraoral scanner before and after tooth preparation. No studies with similar research objectives can be found in the literature about the Evo I.O. Scan Fussen S 600. Only a few studies have been conducted on the Virtuo Vivo intraoral scanner—VV (Dental Wings)—but with different objectives, mainly involving implant-prosthetic applications [30,31]. Obtaining the correct reference points to perform the measurements correctly was not easy in all cases. It was problematic in the case of MeshLab software (Version: 2023.12). The values obtained with this software were significantly different from the reference values. For the analysis of the data, the values from Zeiss Inspect software (Version: 2023.3.0.969) were used. All the values obtained from the digital measurements were higher than the reference values (Table 1 and Table 2). Minor discrepancies were found in the A2-L2 and A1-B1 distances in the upper models compared to the reference model. The lowest differences for all lower models were obtained for the a1-b1 distance, except for the non-prepared models scanned with the two intraoral scanners, where differences were not found related to the reference model (Table 1 and Table 2). The discrepancies during the scan became higher as the scanning head moved away from the starting point.

The scanning path used can negatively or positively influence the accuracy of the scans. Intraoral scanners do not have a standardized scanning protocol; for these reasons, the scanning procedure has been carried out according to the manufacturer’s recommendation for each scanner, but for the lower model, the direction was inversed. Both manufacturers of the two intraoral scanners recommend the same scanning sequence, which was implemented during this study. This scanning sequence has been shown to be most effective for other scanners as well [32]. The manufacturer’s recommended path can lead to discrepancies because it is not scientifically supported. Despite the scanning path’s reverse direction, non-prepared lower models’ values were similar. In this case, the reverse scan direction was more favorable. Other directions recommended by other authors can be more efficient.

An acceptable scanning accuracy range of less than 100–120 µm has been recommended by several authors in the literature [33,34,35,36,37]. In this study, we obtained lower discrepancies than the recommended range only from the lower digital models for the a1-b1 distance (VV np; NOT np, p; IOS p) and the b2-c distance (VV np, IOS np) (Table 2). A study by Kernen et al. demonstrated that laboratory scanners are more accurate than the intraoral scanners used in dental offices [38]. Sason et al. conducted a study that concluded that the intraoral scanner used in the study had higher accuracy than the laboratory scanner used for the digitization of models obtained by conventional impression with additional silicone materials [39]. These conclusions, however, may be due to mistakes during the impression or the making of working casts, due to volumetric changes in the impression of impression materials or gypsum. A similar in vitro study also shows that digital models resulting from intraoral scanning have similar or better accuracy than digital models obtained from the digitization of casts as a result of conventional impressions [40].

Other researchers have demonstrated differences in the accuracy of different intraoral scanners depending on the lighting conditions of the oral cavity [41]. Already in 2008, it was shown that darkness provides optimal conditions for intraoral scanning [42]. The most optimal ambient lighting for intraoral scanning is 3900 K temperature and 500 lux illumination, typical for dental offices. Very intense illumination increases scanning time [43]. In the case of our study, the full-arch scans of the simulator models were performed in vitro under similar conditions, but still in a different environment than the oral cavity, and this can influence the obtained results.

Many studies have investigated the accuracy of full-arch scans [44,45], but others demonstrated that scan accuracy is better in the frontal areas of the arches, with larger differences in the posterior areas [46]. In our case, the lowest values were obtained in the transversal plane at the most posterior reference points, which contradicts with these studies. The differences between measurements in our study may also be due to the full-arch scanning protocol. Digital scanners can be sensitive to decalibration and record distorted information. The periodic calibration of these instruments results in the accurate recording of the prosthetic field, as demonstrated by Rehmann et al. in their study [47]. The authors of this study recommend constant maintenance of the scanners for their optimal functioning. One of the intraoral scanners used in this study was self-calibrating. Pre-scan calibration was not possible, which could lead to errors during the scanning procedures. Other studies from literature examined the scanning accuracy related to the existence and size of edentulous areas in the arches [48,49]. However, there are no studies on accuracy differences for full-arch scans of complete arches with intact versus integral arches with fewer or more teeth prepared for restoration. This is particularly relevant in the case of fixed restorative prosthodontic treatments involving tooth preparation. Our results demonstrated that a higher number of prepared teeth can modify the scanning accuracy. For all the scanners, the dimensional differences between the prepared and non-prepared lower models were higher and more frequent than for the uppers. The discrepancy between the values from the laboratory upper and lower models and those from the two intraoral scanners was substantial (Table A4). The differences were more evident for the upper non-prepared models’ diagonal values and the prepared models’ diagonal and transversal values. No discrepancy was found for most of the values from the non-prepared and prepared models of the two intraoral scanners. The differences were evident on the A1-B1 transversal and posterior distance (higher on the prepared models), respectively, and on the R3-L3 transversal, median (non-prepared), and B2-R2 (prepared) diagonal distances. The values for the lower models are more different, probably due to the larger number of prepared teeth. Only the a2-l2 diagonal distance is similar on all models for all scanners.

The results obtained have clinical relevance in the context of prosthetic treatments, particularly in instances where a digital workflow is employed to generate the models necessary for making fixed restorations on previously prepared teeth. The accuracy of the scanning process has a significant impact, particularly for chair-side treatments. It is important to examine the performance of these simpler and lower-priced scanners, as their clinical applicability with favorable results would also open new perspectives for clinicians who do not have the financial possibilities to purchase top-of-the-range scanners but aspire to implement digital workflow. This study provides information for specialists in the field about the scanning accuracy of the three used scanners. The results can highlight errors during the full-arch scans of non-prepared and prepared teeth according to their number and location on the dental arches. Based on the information obtained from this study, it is possible to assess the regions where scanning accuracy is lower and conclude how the position and number of prepared scanned teeth influence the effectiveness of using this modern method for fixed prosthetic restorations. The data from this study can allow clinicians to choose one of the tested scanners and adapt its use to different clinical situations depending on the number and location of the teeth that need to be restored. This study can increase the quality of restorations and the whole rehabilitation procedure.

In terms of the limitations of the study, the number of the scanners compared is small. The two intraoral scanners used are not among the most popular, are simplier, and can only be used for impressions without other applications or digital design functions. The scans were carried out by a single experienced operator, and other people with a different level of experience may give different results. The fatigue of the person who performs the scans can affect the accuracy. In our study, the same person did the scanning with the intraoral scanners. All the scans were obtained on the same day for all the scanners. The reference values were obtained using manual measurements with the digital caliper, which can lead to errors, causing discrepancies in the obtained digital models. The results obtained for different areas of the dental arches could be clarified with greater clinical applicability by using a larger number of models and a variety of locations and number of the prepared teeth. The simulator models were not scanned under oral cavity conditions, but under the light source of the dental practice or laboratory. The values obtained in this way in terms of accuracy may be different from those obtained from scans performed in the oral cavity and may differ from reality because no standardization instrument was used for the measurements.

Considering these limitations and the requirements of current practice, further studies are needed to demonstrate the need for the use of digital impressions to reduce effort, working time, and to minimize human and material errors in rehabilitation procedures. Therefore, we will carry out new studies to verify the accuracy of full-arch scanning using landmark standardization devices. Different scanning paths will be examined, defining the most suitable for these scanners. We will compare the results with those obtained from conventional impressions on a larger number of models and also in vivo.

## 5. Conclusions

Based on the findings of this study, the following conclusions were drawn:

- The accuracy of the scans can be influenced by the number of prepared teeth and their position on the dental arches. Distortions may occur on dental arches with multiple preparations, as in our study in the case of the lower arch.
- The digital models are distorted in full-arch scans, with the level of distortion being related to the scanning distance from the starting point.
- The manufacturer’s recommended scanning path may not always yield the most accurate results for intraoral scanners. Scientific research should guide the selection of the appropriate scanning path for each scanner, as was the case with the intraoral scanners used during this research.
- Self-calibrating systems may reduce accuracy; therefore, regular calibration is essential for obtaining realistic parameters during the scanning process.

## Figures and Tables

**Table 1:** The discrepancy between all maxillary distances and references model.

	Discrepancy						Theoretical Mean	*p* Value
	VV np	VV p	NOT np	NOT p	IOS np	IOS p		
A1-B1	0.3075	0.5445	0.4465	0.671	0.5045	0.2975	50.71	<0.0001
R3-L3	0.9015	1.164	1.103	1.313	1.103	1.199	33.7	
R1-L1	1.245	1.158	1.315	1.108	1.201	1.084	24.7	
A2-L2	0.265	0.3665	0.631	0.576	0.360	0.3105	46.54	
A2-D	2.490	2.330	1.308	1.334	2.504	1.994	50.76	
B2-R2	0.6945	0.7630	0.7295	0.898	0.685	0.752	46.54	
B2-C	3.278	1.316	1.023	0.979	1.691	3.941	50.76	

**Table 2:** The discrepancy between all mandibular distances and references model.

	Discrepancy						Theoretical Mean	*p* Value
	VV np	VV p	NOT np	NOT p	IOS np	IOS p		
a1-b1	0.049	0.339	0.111	0.0995	−0.209	0.046	45.18	<0.05
r3-l3	1.375	1.036	1.147	1.280	1.148	0.9965	30.46	
r1-l1	0.4901	0.6031	0.4821	0.7351	0.6216	0.6211	20.60	
a2-l2	1.199	1.204	1.420	1.511	1.171	1.241	44.80	
a2-d	0.4915	0.4555	0.437	0.5965	1.051	0.604	58.46	
b2-r2	0.5465	0.616	0.682	0.7565	0.5355	0.4955	44.80	
b2-c	0.0295 ns	0.409	0.267	0.5565	0.059 ns	0.4795	58.46	

ns—not significant statistically.

## Data Availability

The dataset analyzed during this article is available from the first author on request.

## Appendix A

**Table A1:** The descriptive statistic for all maxillary virtual models.

		Minimum (min)	Median (M)	Maximum (Max)	Mean (m)	Std. Deviation (SD)	Lower 95% CI of Mean	Upper 95% CI of Mean
VV np	A1-B1	50.74	51.02	51.45	51.04	0.2634	50.97	51.12
	R3-L3	34.25	34.6	34.96	34.58	0.2556	34.51	34.66
	R1-L1	25.35	25.94	26.39	25.89	0.3727	25.78	26
	A2-L2	46.4	46.81	47.06	46.77	0.2199	46.7	46.83
	A2-D	51.5	53.25	54.48	53.16	1.006	52.86	53.45
	B2-R2	46.86	47.23	47.46	47.2	0.1805	47.14	47.25
	B2-C	50.57	54.03	55.11	53.59	1.472	53.16	54.02
VV p	A1-B1	50.97	51.25	51.81	51.37	0.2579	51.29	51.44
	R3-L3	34.26	34.86	35.1	34.77	0.3017	34.69	34.86
	R1-L1	25.35	25.86	26.49	25.86	0.3473	25.76	25.96
	A2-L2	46.38	46.9	47.4	46.9	0.3367	46.8	47
	A2-D	51.6	53.09	53.94	53.01	0.7064	52.8	53.21
	B2-R2	47.1	47.3	47.68	47.34	0.1644	47.29	47.38
	B2-C	50.6	52.07	55.58	52.93	1.775	52.42	53.45
NOT np	A1-B1	50.99	51.15	51.45	51.17	0.1382	51.13	51.21
	R3-L3	33.87	34.8	35.01	34.68	0.3528	34.58	34.79
	R1-L1	25.86	26.01	26.08	25.98	0.0884	25.95	26
	A2-L2	46.85	47.17	47.47	47.19	0.1811	47.14	47.24
	A2-D	51.77	52.06	52.61	52.08	0.2538	52.01	52.15
	B2-R2	47.03	47.27	47.73	47.31	0.2218	47.24	47.37
	B2-C	51.46	51.78	52.01	51.73	0.1892	51.67	51.78
NOT p	A1-B1	51.23	51.38	51.53	51.38	0.0886	51.36	51.41
	R3-L3	34.75	35.01	35.3	35.04	0.175	34.99	35.09
	R1-L1	25.14	25.81	26.14	25.73	0.3412	25.63	25.83
	A2-L2	46.97	47.11	47.28	47.13	0.1045	47.1	47.16
	A2-D	51.83	52.09	52.43	52.12	0.2016	52.06	52.18
	B2-R2	47.22	47.44	47.65	47.43	0.1529	47.38	47.47
	B2-C	51.38	51.74	51.98	51.72	0.2035	51.66	51.78
IOS np	A1-B1	50.99	51.21	51.57	51.23	0.1996	51.17	51.29
	R3-L3	34.32	34.8	35.05	34.76	0.2783	34.68	34.84
	R1-L1	24.96	25.9	26.19	25.84	0.3567	25.74	25.94
	A2-L2	46.55	46.84	47.15	46.85	0.2018	46.79	46.91
	A2-D	52.58	53.26	54.23	53.35	0.5459	53.19	53.51
	B2-R2	47.08	47.22	47.3	47.21	0.0666	47.19	47.23
	B2-C	51.51	52.45	55.2	52.82	1.202	52.47	53.17
IOS p	A1-B1	50.39	51.01	51.29	50.9	0.3403	50.8	51
	R3-L3	34.41	34.9	35.15	34.85	0.2473	34.78	34.93
	R1-L1	25.43	25.78	26.21	25.83	0.2474	25.75	25.9
	A2-L2	46.59	46.85	47.28	46.9	0.26	46.82	46.97
	A2-D	51.48	52.75	53.69	52.71	0.6907	52.51	52.91
	B2-R2	46.84	47.29	47.37	47.17	0.2051	47.11	47.23
	B2-C	51.25	54.25	54.84	53.51	1.418	53.1	53.93

V np: Virtuo Vivo scanner, non-prepared model, VV p: Virtuo Vivo scanner, prepared model; NOT np: Neway Open Tech scanner, non-prepared model; NOT p: Neway Open Tech scanner, prepared model; IOS np: Evo I.O Scan Fussen scanner, non-prepared model; IOS p: Evo I.O Scan Fussen scanner, prepared model.

**Table A2:** The descriptive statistic for all mandibular virtual models.

		Minimum (min)	Median (M)	Maximum (Max)	Mean (m)	Std. Deviation (SD)	Lower 95% CI of Mean	Upper 95% CI of Mean
VV np	a1-b1	45.04	45.23	45.7	45.28	0.213	45.22	45.35
	r3-l3	31.48	31.83	32.25	31.83	0.2219	31.77	31.9
	r1-l1	20.9	21.09	21.23	21.08	0.1029	21.05	21.11
	a2-l2	45.93	45.99	46.18	46.03	0.0808	46.01	46.06
	a2-d	48.68	48.95	48.98	48.89	0.1014	48.86	48.92
	b2-r2	45.14	45.34	45.47	45.31	0.1214	45.28	45.35
	b2-c	47.96	48.49	48.81	48.45	0.2963	48.37	48.54
VV p	a1-b1	45.18	45.52	45.84	45.52	0.221	45.45	45.58
	r3-l3	31	31.49	31.56	31.41	0.1778	31.36	31.46
	r1-l1	21.15	21.2	21.4	21.25	0.096	21.22	21.28
	a2-l2	45.9	46	46.11	46.01	0.0572	45.99	46.03
	a2-d	48.25	48.92	48.98	48.85	0.23	48.78	48.92
	b2-r2	45.27	45.41	45.7	45.46	0.1451	45.42	45.5
	b2-c	48.8	48.87	48.96	48.87	0.0469	48.86	48.89
NOT np	a1-b1	45.05	45.29	45.63	45.31	0.1857	45.25	45.36
	r3-l3	31.51	31.6	31.7	31.6	0.0736	31.58	31.62
	r1-l1	20.99	21.08	21.3	21.1	0.1029	21.07	21.13
	a2-l2	46	46.22	46.41	46.21	0.1274	46.18	46.25
	a2-d	48.86	48.9	48.97	48.91	0.04	48.9	48.92
	b2-r2	45.34	45.48	45.6	45.47	0.0858	45.45	45.5
	b2-c	48.56	48.73	48.98	48.75	0.152	48.7	48.79
NOT p	a1-b1	45.11	45.28	45.44	45.28	0.1233	45.24	45.32
	r3-l3	31.63	31.74	31.78	31.73	0.0443	31.71	31.74
	r1-l1	21.14	21.34	21.46	21.33	0.0937	21.31	21.36
	a2-l2	46.1	46.31	46.48	46.28	0.1217	46.25	46.32
	a2-d	48.89	49.06	49.18	49.04	0.0894	49.01	49.06
	b2-r2	45.36	45.55	45.64	45.52	0.1016	45.49	45.55
	b2-c	48.78	49.02	49.1	48.99	0.1053	48.96	49.02
IOS np	a1-b1	44.62	44.97	45.24	44.99	0.2016	44.93	45.05
	r3-l3	31.2	31.61	31.98	31.6	0.2575	31.52	31.67
	r1-l1	20.93	21.22	21.5	21.22	0.1582	21.17	21.26
	a2-l2	45.67	45.97	46.12	45.92	0.1609	45.87	45.96
	a2-d	49.02	49.51	50.97	49.72	0.6737	49.53	49.92
	b2-r2	45.12	45.33	45.41	45.28	0.1205	45.25	45.32
	b2-c	48.07	48.52	48.81	48.5	0.2353	48.43	48.57
IOS p	a1-b1	45.11	45.23	45.86	45.29	0.2243	45.22	45.35
	r3-l3	31.11	31.45	31.93	31.49	0.26	31.42	31.57
	r1-l1	21.03	21.22	21.58	21.25	0.159	21.2	21.3
	a2-l2	45.54	46.04	46.19	46	0.1874	45.94	46.05
	a2-d	48.88	49.07	49.15	49.03	0.0848	49.01	49.06
	b2-r2	44.97	45.3	45.63	45.33	0.193	45.28	45.39
	b2-c	48.8	48.94	49.1	48.94	0.1011	48.91	48.97

V np: Virtuo Vivo scanner, non-prepared model, VV p: Virtuo Vivo scanner, prepared model; NOT np: Neway Open Tech scanner, non-prepared model; NOT p: Neway Open Tech scanner, prepared model; IOS np: Evo I.O Scan Fussen scanner, non-prepared model; IOS p: Evo I.O Scan Fussen scanner, prepared model.

## Appendix B

**Table A3:** The maxillary measured distances in scanning patters using the Kruskal–Wallis test with Dunn’s post hoc test.

		VV np	VV p	NOT np	NOT p	IOS np	IOS p
VV np	A1-B1	-	<0.0001	ns	<0.0001	0.0404	ns
	R3-L3	-	0.0029	ns	<0.0001	0.0129	<0.0001
	R1-L1	-	ns	ns	ns	ns	ns
	A2-L2	-	ns	<0.0001	<0.0001	ns	ns
	A2-D	-	ns	<0.0001	<0.0001	ns	ns
	B2-R2	-	0.011	ns	0.011	ns	ns
	B2-C	-		<0.0001	<0.0001	ns	ns
VV p	A1-B1	<0.0001	-	0.0031	ns	ns	<0.0001
	R3-L3	0.0029	-	ns	0.0003	ns	ns
	R1-L1	ns	-	ns	ns	ns	ns
	A2-L2	ns	-	<0.0001	0.0006	ns	ns
	A2-D	ns	-	<0.0001	<0.0001	ns	ns
	B2-R2	0.011	-	ns	ns	0.028	0.028
	B2-C	ns	-	<0.0001	<0.0001	ns	ns
NOT np	A1-B1	ns	0.0031	-	<0.0001	ns	ns
	R3-L3	ns	ns	-	<0.0001	ns	ns
	R1-L1	ns	ns	-	0.0019	ns	ns
	A2-L2	<0.0001	<0.0001	-	ns	<0.0001	<0.0001
	A2-D	<0.0001	<0.0001	-	ns	<0.0001	0.0002
	B2-R2	ns	ns	-	ns	0.016	ns
	B2-C	<0.0001	<0.0001	-	ns	<0.0001	<0.0001
NOT p	A1-B1	<0.0001	ns	<0.0001	-	0.0014	<0.0001
	R3-L3	<0.0001	0.0003	<0.0001	-	<0.0001	0.0259
	R1-L1	ns	ns	0.0019	-	ns	ns
	A2-L2	<0.0001	<0.0001	ns	-	<0.0001	0.0004
	A2-D	<0.0001	<0.0001	ns	-	<0.0001	0.0007
	B2-R2	0.011	ns	ns	-	<0.0001	ns
	B2-C	<0.0001	<0.0001	ns	-	<0.0001	<0.0001
IOS np	A1-B1	0.0404	ns	ns	0.0014	-	0.0001
	R3-L3	0.0129	ns	ns	<0.0001	-	ns
	R1-L1	ns	ns	ns	ns	-	ns
	A2-L2	ns	ns	<0.0001	<0.0001	-	ns
	A2-D	ns	ns	<0.0001	<0.0001	-	0.0022
	B2-R2	ns	0.028	0.016	<0.0001	-	ns
	B2-C	ns	ns	<0.0001	<0.0001	-	ns
IOS p	A1-B1	ns	<0.0001	ns	<0.0001	0.0001	-
	R3-L3	<0.0001	ns	ns	<0.0001	ns	-
	R1-L1	ns	ns	ns	ns	ns	-
	A2-L2	ns	ns	<0.0001	0.0004	ns	-
	A2-D	ns	0.0002	0.0007	ns	0.0022	-
	B2-R2	ns	<0.0001	ns	ns	ns	-
	B2-C	ns	ns	<0.0001	<0.0001	ns	-

ns—not significant statistically.

**Table A4:** The mandibular measured distances in scanning patters using the Kruskal–Wallis test with Dunn’s post hoc test.

		VV np	VV p	NOT np	NOT p	IOS np	IOS p
VV np	a1-b1	-	<0.0001	ns	ns	<0.0001	
	r3-l3	-	<0.0001	<0.0001	ns	<0.0001	<0.0001
	r1-l1	-	<0.0001	ns	<0.0001	0.0003	<0.0001
	a2-l2	-	ns	<0.0001	<0.0001	ns	ns
	a2-d	-	ns	ns	<0.0001	<0.0001	<0.0001
	b2-r2	-	<0.0001	<0.0001	<0.0001	ns	ns
	b2-c	-	<0.0001	0.0062	<0.0001	ns	0.0062
VV p	a1-b1	<0.0001	-	0.003	<0.0001	<0.0001	<0.0001
	r3-l3	<0.0001	-	0.0062	<0.0001	0.0013	ns
	r1-l1	<0.0001	-	<0.0001	ns	ns	ns
	a2-l2	ns	-	<0.0001	<0.0001	ns	ns
	a2-d	ns	-	ns	<0.0001	<0.0001	<0.0001
	b2-r2	<0.0001	-	ns	ns	<0.0001	0.0177
	b2-c	<0.0001	-	0.0233	0.0206	<0.0001	ns
NOT np	a1-b1	ns	0.003	-	ns	<0.0001	ns
	r3-l3	<0.0001	0.0062	-	0.0037	ns	ns
	r1-l1	ns	<0.0001	-	<0.0001	0.0013	<0.0001
	a2-l2	<0.0001	<0.0001	-	ns	<0.0001	<0.0001
	a2-d	ns	ns	-	<0.0001	<0.0001	<0.0001
	b2-r2	<0.0001	ns	-	ns	<0.0001	0.0003
	b2-c	0.0062	0.0233	-	<0.0001	0.0068	<0.0001
NOT p	a1-b1	ns	<0.0001	ns	-	<0.0001	ns
	r3-l3	ns	<0.0001	0.0037	-	0.0164	<0.0001
	r1-l1	<0.0001	ns	<0.0001	-	0.0003	0.0074
	a2-l2	<0.0001	<0.0001	ns	-	<0.0001	<0.0001
	a2-d	<0.0001	<0.0001	<0.0001	-	<0.0001	ns
	b2-r2	<0.0001	ns	ns	-	<0.0001	<0.0001
	b2-c	<0.0001	0.0206	<0.0001	-	<0.0001	ns
IOS np	a1-b1	<0.0001	<0.0001	<0.0001	<0.0001	-	<0.0001
	r3-l3	<0.0001	0.0013	ns	0.0164	-	ns
	r1-l1	0.0003	ns	0.0013	0.0003	-	ns
	a2-l2	ns	ns	<0.0001	<0.0001	-	ns
	a2-d	<0.0001	<0.0001	<0.0001	<0.0001	-	<0.0001
	b2-r2	ns	<0.0001	<0.0001	<0.0001	-	
	b2-c	ns	<0.0001	0.0068	<0.0001	-	<0.0001
IOS p	a1-b1	ns	<0.0001	ns	ns	<0.0001	-
	r3-l3	<0.0001	ns	ns	<0.0001	ns	-
	r1-l1	<0.0001	ns	<0.0001	0.0074	ns	-
	a2-l2	ns	ns	<0.0001	<0.0001	ns	-
	a2-d	<0.0001	<0.0001	<0.0001	ns	<0.0001	-
	b2-r2	ns	0.0177	0.0003	<0.0001	ns	-
	b2-c	0.0062	ns	<0.0001	ns	<0.0001	-

ns—not significant statistically.
